# Supplementary material for: Mycobacterium tuberculosis growth arrest on propionate at acidic pH is suppressed by mutations in phoPR and pyrazinamide treatment
Source: mBio. 2025 Nov 28;17(1):e02955-25. doi: 10.1128/mbio.02955-25 (PMC12802220; doi:10.1128/mbio.02955-25)
Supplement: Supplemental Material — Table S1 and Figures S1 to S10. [file mbio.02955-25-s0001.pdf]

Supplemental Table and Figures

Mycobacterium tuberculosis **growth arrest on propionate at acidic pH is suppressed by mutations in *phoPR* and pyrazinamide treatment**

Heather M. Murdoch[1,2], Shelby J. Dechow[1,2], Bassel J. Abdalla[1] and Robert B. Abramovitch[1]

[1]Department of Microbiology, Genetics & Immunology, Michigan State University, East Lansing, MI,48824

[2]Co-first authors contributed equally to this study.

Supplemental Table

Table S1: Transposon Mutant Propionate Selection Summary

Supplemental Figure Legends

Figure S1. Bacterial Growth and viability of WT, Δ*phoPR* mutant, and complemented strains grown on propionate and treated with MSU-43996 at neutral pH. A) bacterial growth of the three strains on propionate at pH 7.0, showing no significant differences in growth rate. B) bacterial growth of the three strains on propionate at pH 7.0, treated with MSU-43996 or the vehicle, showing no major differences in growth rates based on strain or treatment.

Figure S2. PhoPR restricts growth at acidic pH by diverting carbon from the methyl citrate cycle. A) CFUs from the corresponding experiment in Figure 5A, showing cell death in the ETZ treated icl2 and icl1/2 knockouts. **B)** CFUs of Vitamin B12 treated Mtb strains. **C)** CFUs for figure 5C showing enhanced growth of MSU-43996 treated Mtb. Note, that timepoints are different, but CFUs are from the same experiment. **D**) Vitamin B12 does not impact the growth of strains at neutral pH. A one-way ANOVA was used for the viability experiments, * <0.05, ** <0.01, *** <0.001, **** <0.0001. Experiments were replicated at least twice with similar results.

Figure S3. Function of Icl and PhoPR at neutral pH on propionate. Growth of Mtb strains at pH 7.0 on propionate. ETZ did not impact growth in the *Icl1* or *Icl2* single mutants. The double icl1/2 mutant dies due to propionate toxicity. An unpaired t-test was used between individual groups for the growth curves, * <0.05, ** <0.01, *** <0.001, **** <0.0001. Experiments were replicated at least twice with similar results.

Figure S4. Itaconic acid inhibits enhanced growth of the Δ*phoPR* mutant. Itaconic acid inhibits the ability of the Δ*phoPR* to grow on propionate at pH 5.7. An unpaired t-test was used between individual groups for the growth curves, * <0.05, ** <0.01, *** <0.001, **** <0.0001. Experiments were replicated at least twice with similar results.

Figure S5. Free CoA pools on glycerol as a sole carbon source. During acid growth arrest on glycerol, all three strains maintain relatively low free CoA pools. Two-way ANOVA was used for this analysis, * <0.05, ** <0.01, *** <0.001, **** <0.0001. Experiments were replicated at least twice with similar results.

Figure S6. Vitamin B12 inhibits enhanced growth of WT Mtb treated with PZA. The inhibition of enhanced growth is consistent with PZA inhibiting lipid synthesis to promote growth, as making another lipid synthesis pathway available abolishes enhanced growth. Multiple comparison unpaired t-test was used for the analysis between treatments and the control, and between strains receiving the same treatment, * <0.05, ** <0.01, *** <0.001, **** <0.0001. Experiments were replicated at least twice with similar results.

Figure S7. Vitamin B12 inhibits enhanced growth of WT Mtb treated with PZA. CFU data for OD data presented in Figure S6.

Figure S8. Sensitivity of Mtb to PZA on Pyruvate as a sole carbon source at pH 5.7. PZA treatment does not result in enhanced growth with pyruvate as a sole carbon source.

Figure S9. PZA suppresses acid growth arrest on propionate in Mtb Erdman. To determine if the enhanced growth by PZA is conserved across strains, the PZA treatment experiment from Figure 7 which as conducted in CDC1551, was repeated in Mtb Erdman. Multiple comparison Unpaired t-tests were used for the growth assays, and one-way ANOVA was used for the viability assays, * <0.05, ** <0.01, *** <0.001, **** <0.0001. Experiments were replicated at least twice with similar results.

Figure S10. Sensitivity of Mtb to PZA treatment at pH 7.0. Control experiment for data in Figure 7, showing PZA is not active on Mtb grown in propionate at pH 7.0.

Table S1: Transposon Mutant Propionate Selection Summary

Mutant #	Approx AT or TA site/gene length (nt)	MT number	Rv Number	Gene Number	PDIM Mutation?	Fold Change (Day 14/Day0)
1.1	416/1458	MT0783	Rv0758	*phoR*	None	3.5
1.2	159/1458	MT0783	Rv0758	*phoR*	None	3.3
1.4	311/744	MT0782	Rv0757	*phoP*	None	1.5
2.1	278/1458	MT0783	Rv0758	*phoR*	*ppsE*	2.1
2.4	145/1458	MT0783	Rv0758	*phoR*	*ppsE*	1.9
3.2	145/1458	MT0783	Rv0758	*phoR*	*ppsE*	1.8
3.3	1124/1458	MT0783	Rv0758	*phoR*	*ppsE*	3.6
3.4	416/1458	MT0783	Rv0758	*phoR*	*ppsE*	1.8
4.1	378/1458	MT0783	Rv0758	*phoR*	*ppsE*	1.7
4.2	378/1458	MT0783	Rv0758	*phoR*	*ppsE*	3.5
4.3	416/1458	MT0783	Rv0758	*phoR*	*ppsE*	2.1
4.4	416/1458	MT0783	Rv0758	*phoR*	*ppsE*	2.2

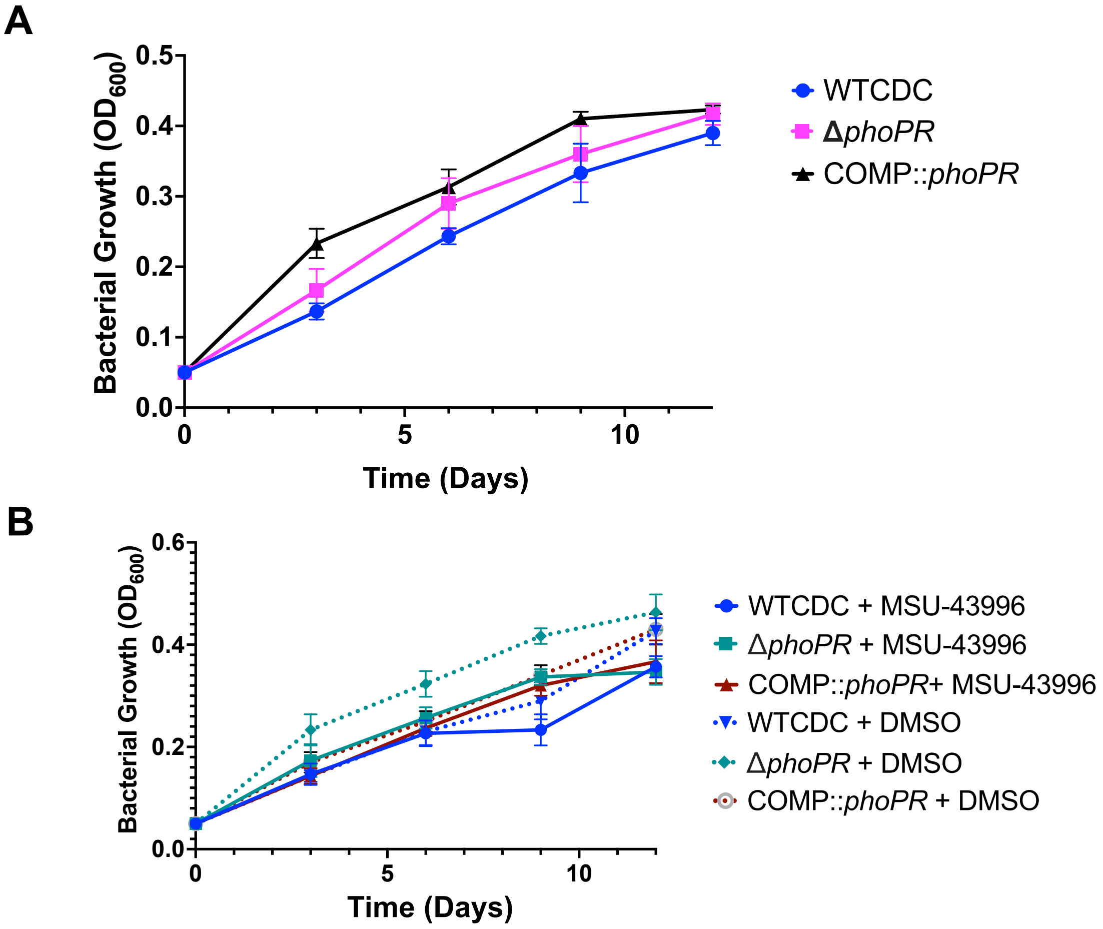

Supplementary Figure 1

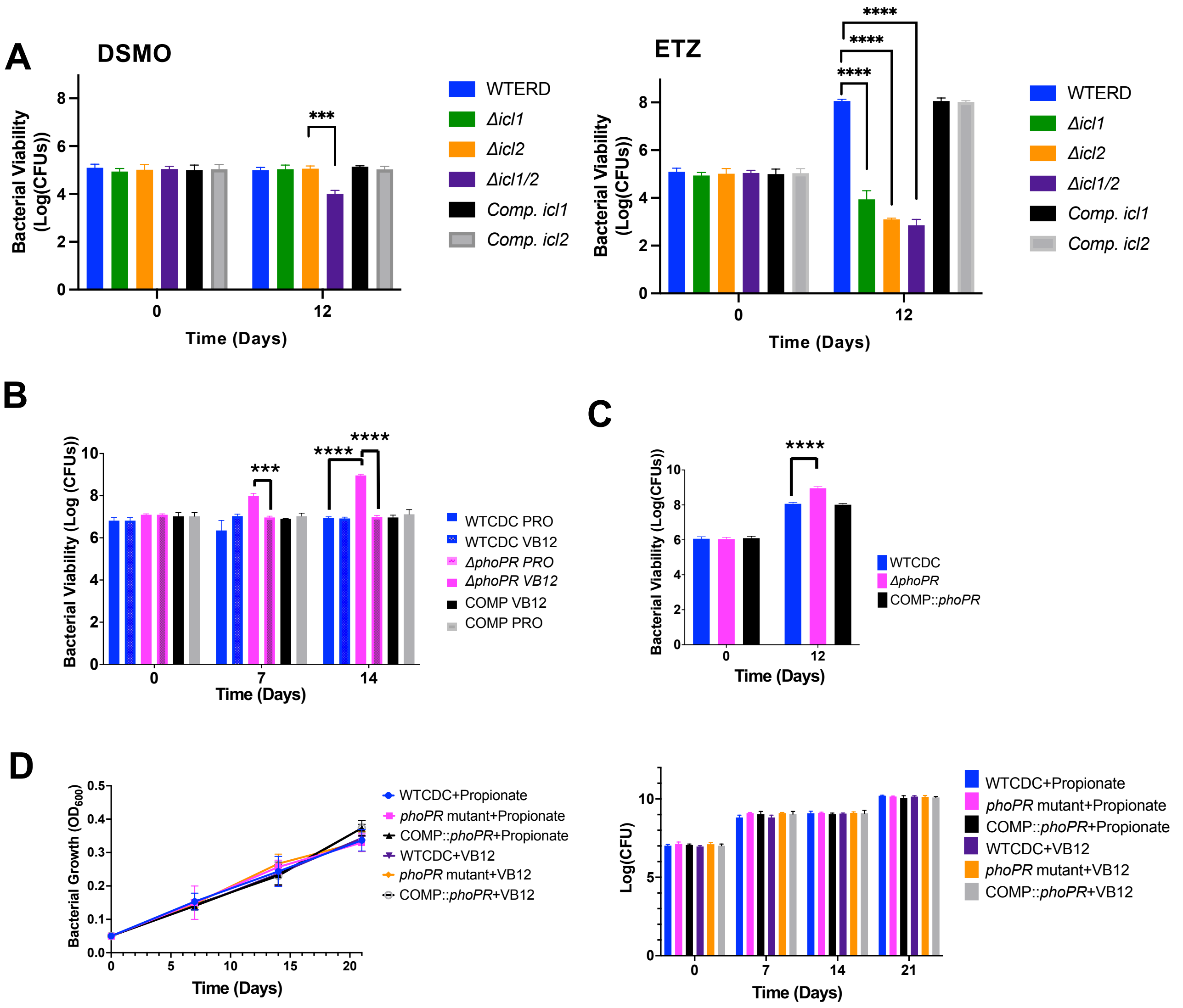

Supplementary Figure 2

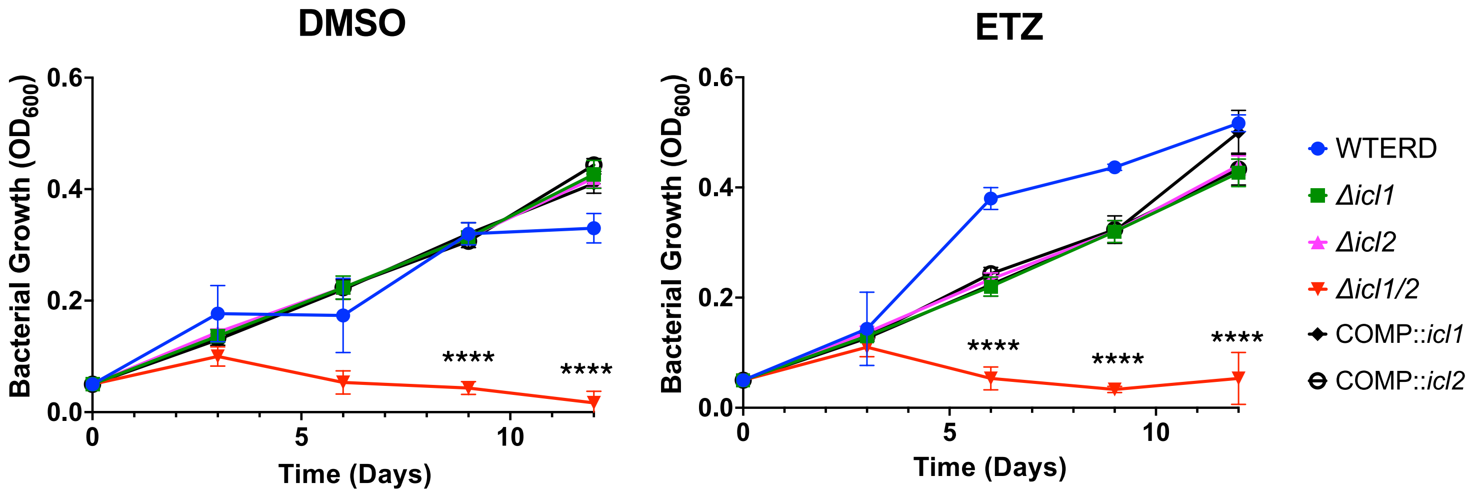

Supplementary Figure 3

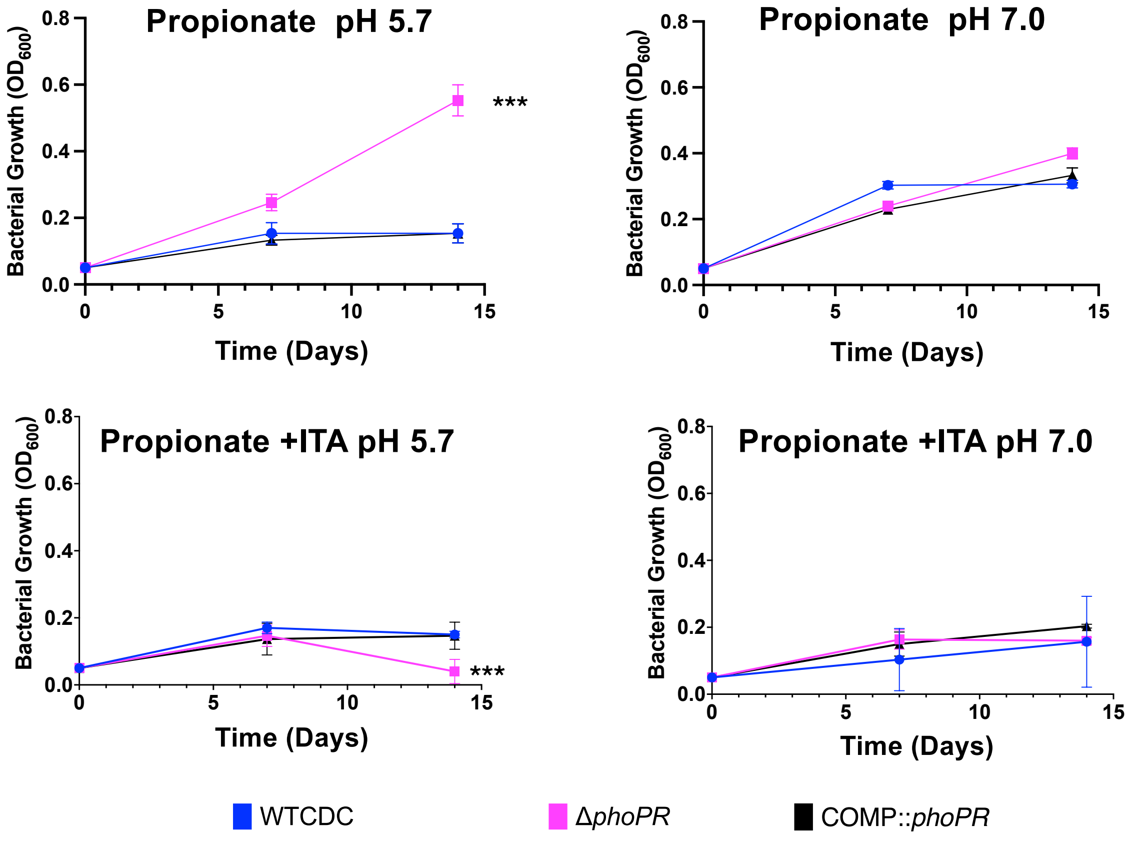

Supplementary Figure 4

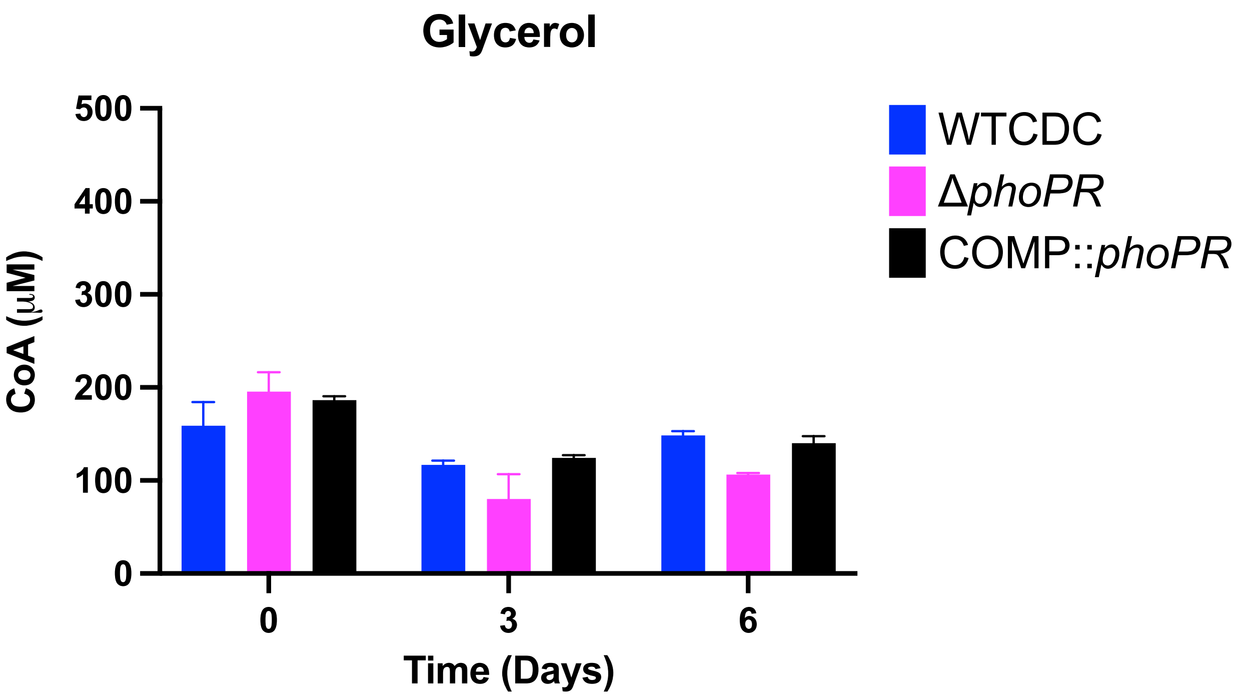

Supplementary Figure 5

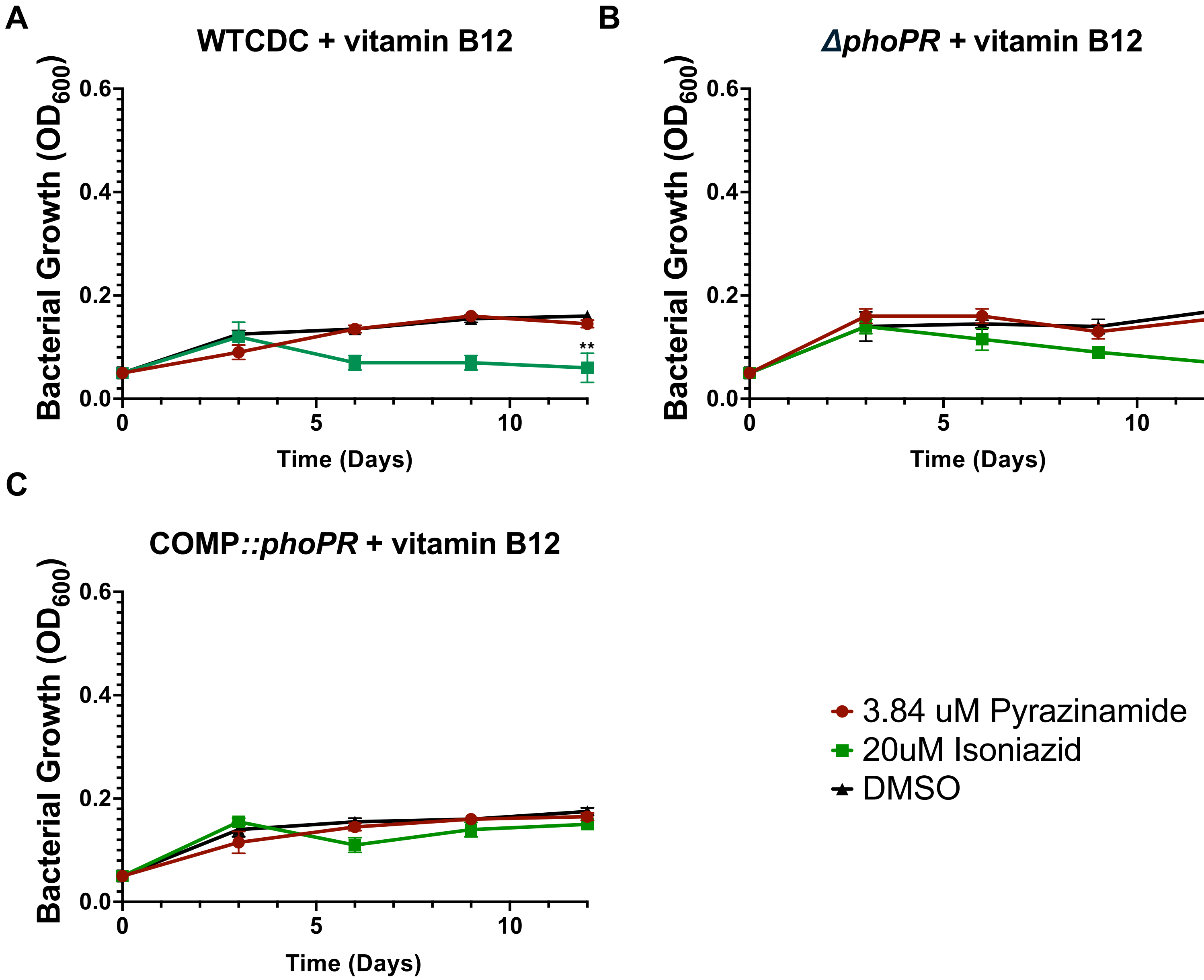

Supplementary Figure 6

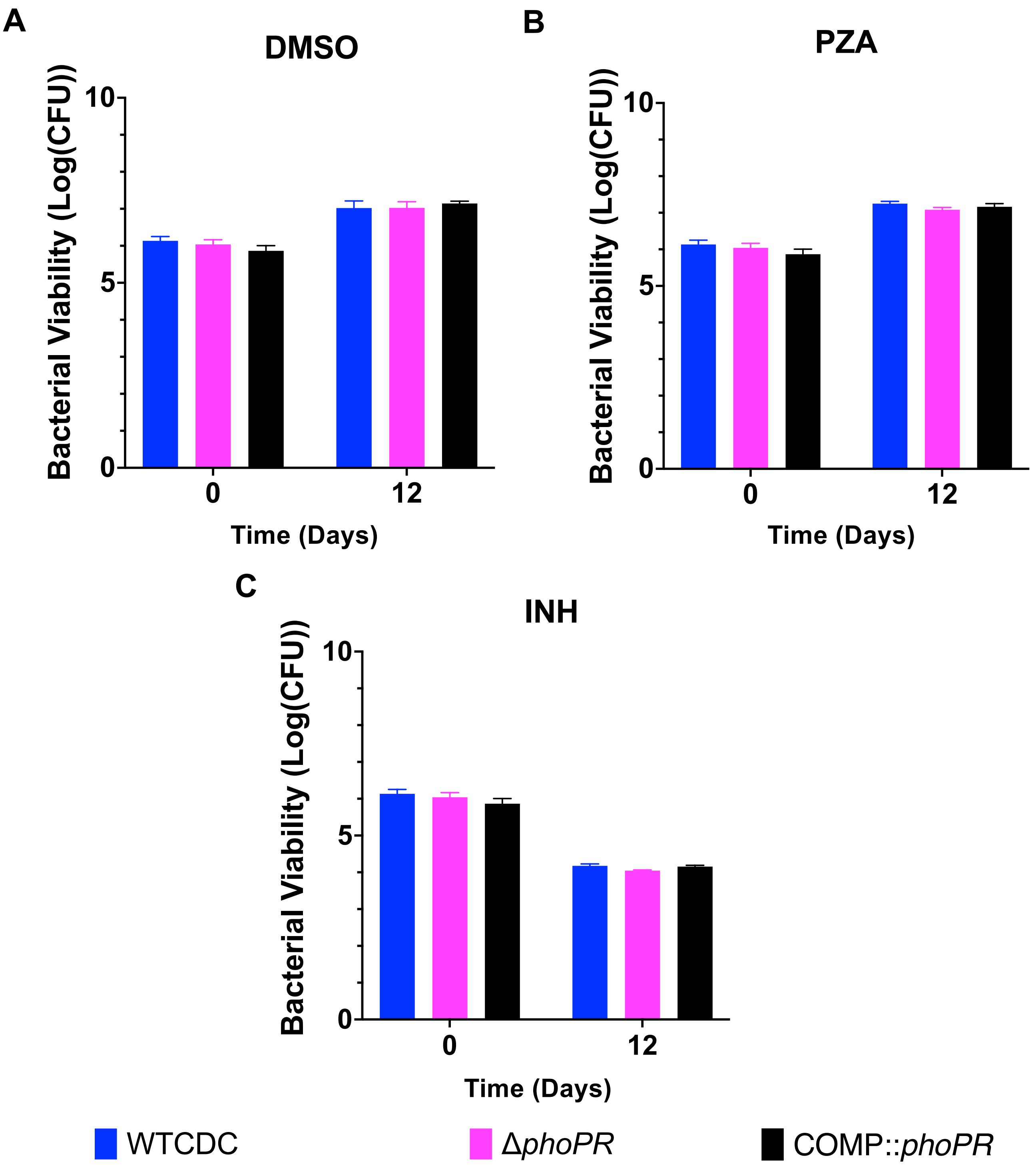

Supplementary Figure 7

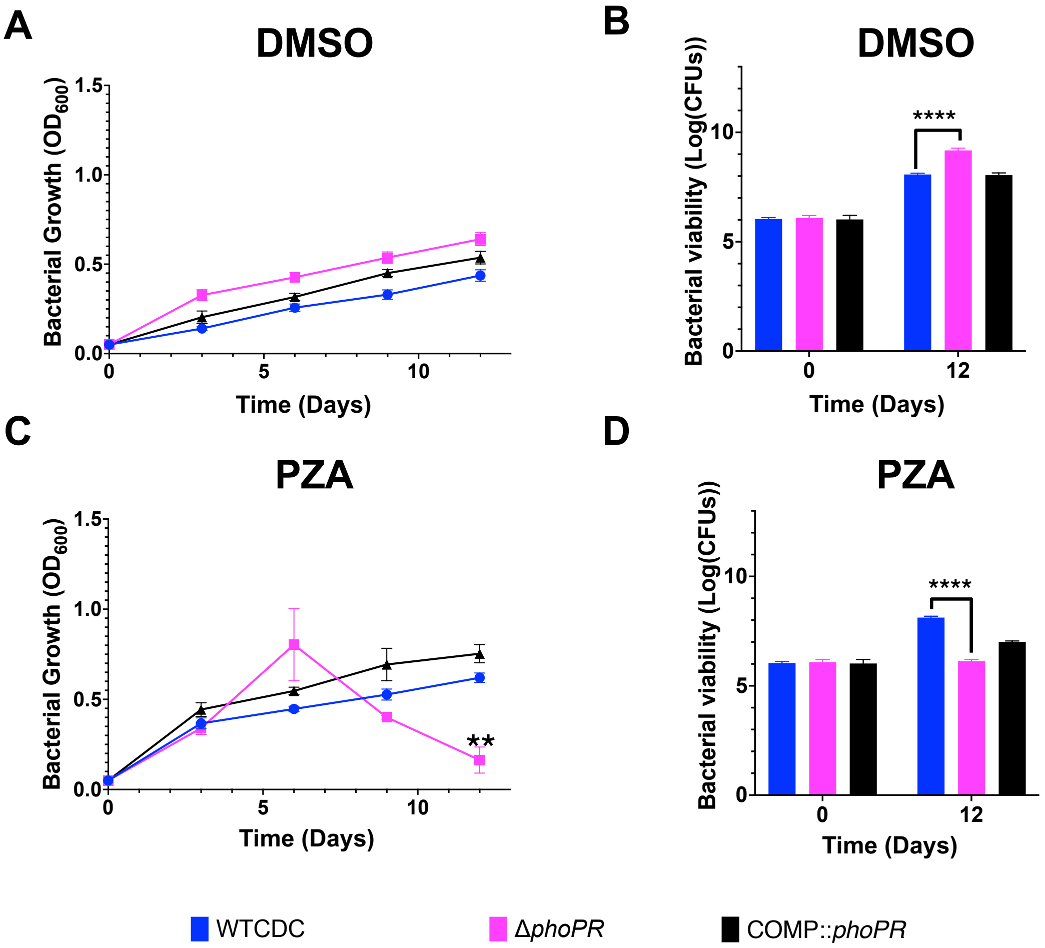

Supplementary Figure 8

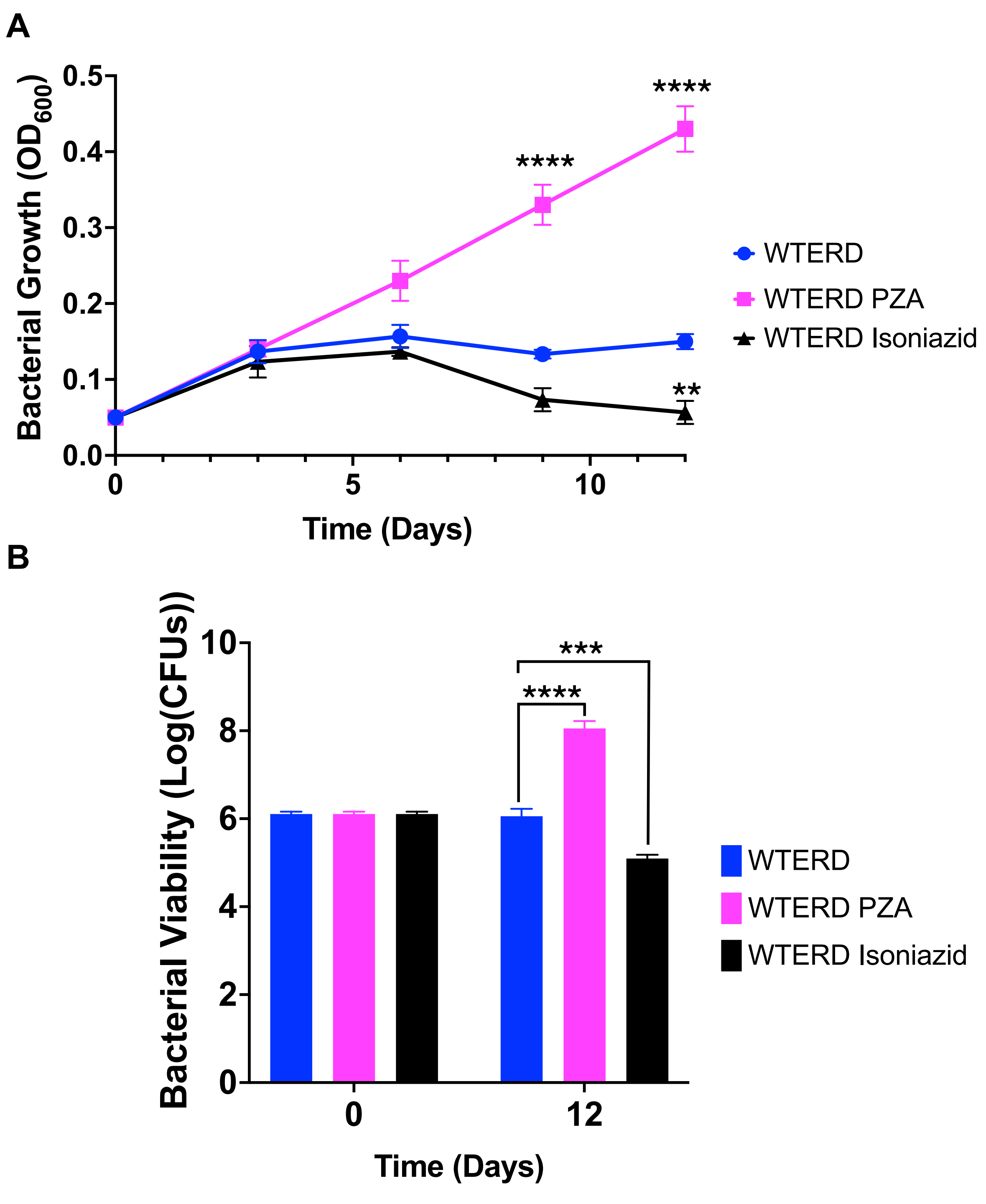

Supplementary Figure 9

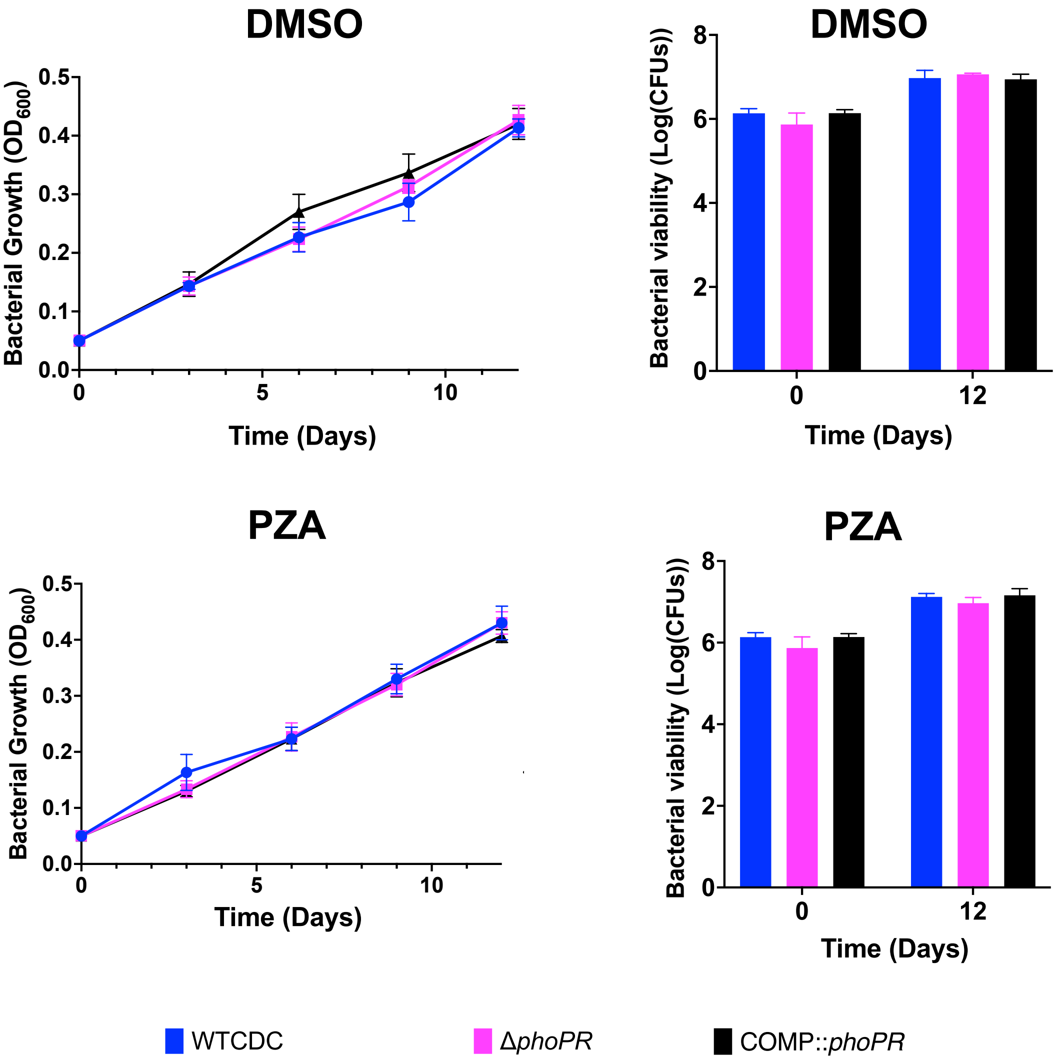

Supplementary Figure 10
